# Supplementary figures and images for: The global scientific research response to the public health emergency of Zika virus infection
Source: PLoS One. 2020 Mar 12;15(3):e0229790. doi: 10.1371/journal.pone.0229790 (PMC7067462; doi:10.1371/journal.pone.0229790)

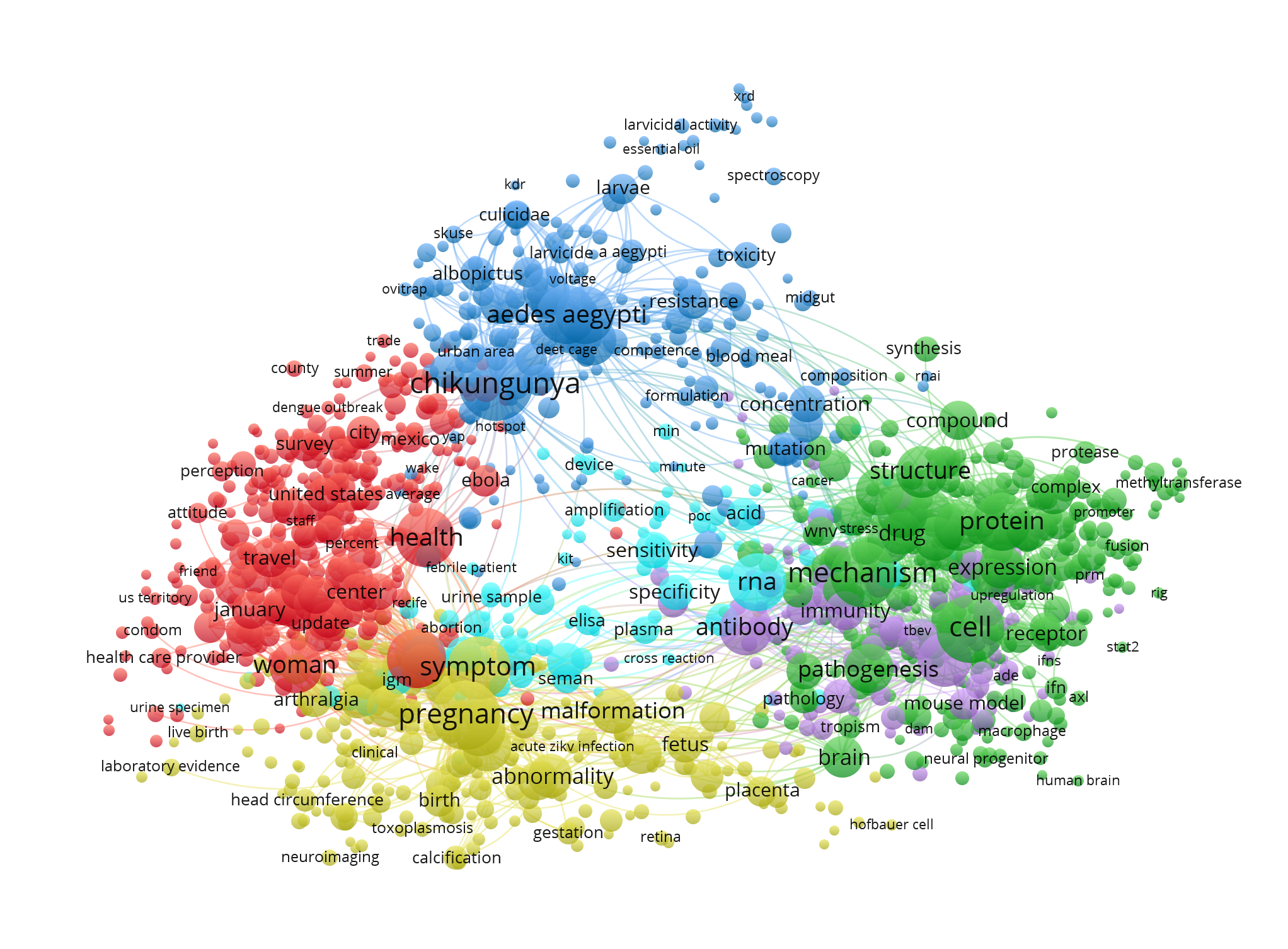

Supplement: S1 Fig — (TIF) [file pone.0229790.s001.tif]

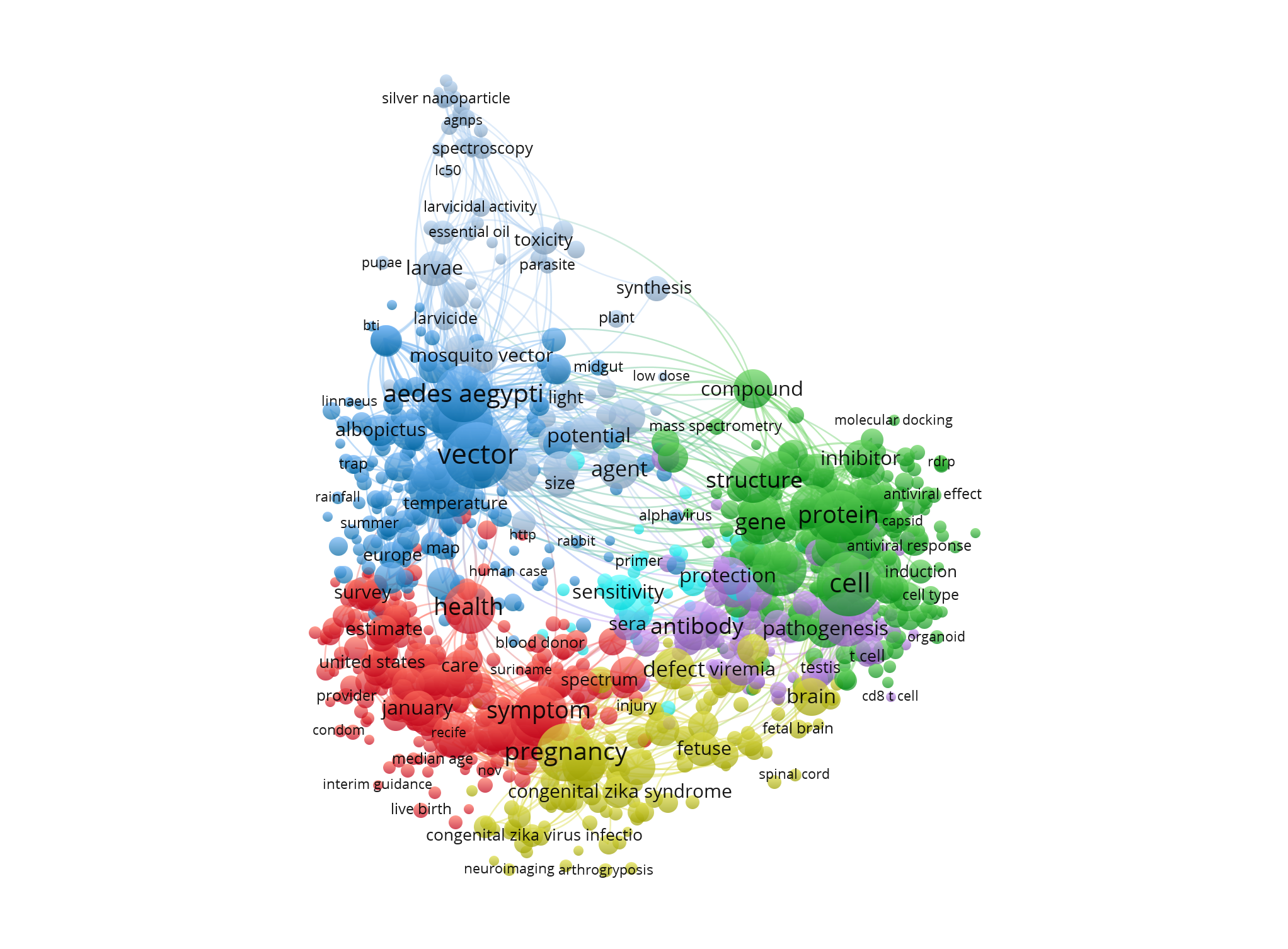

Supplement: S2 Fig — (TIF) [file pone.0229790.s002.tif]

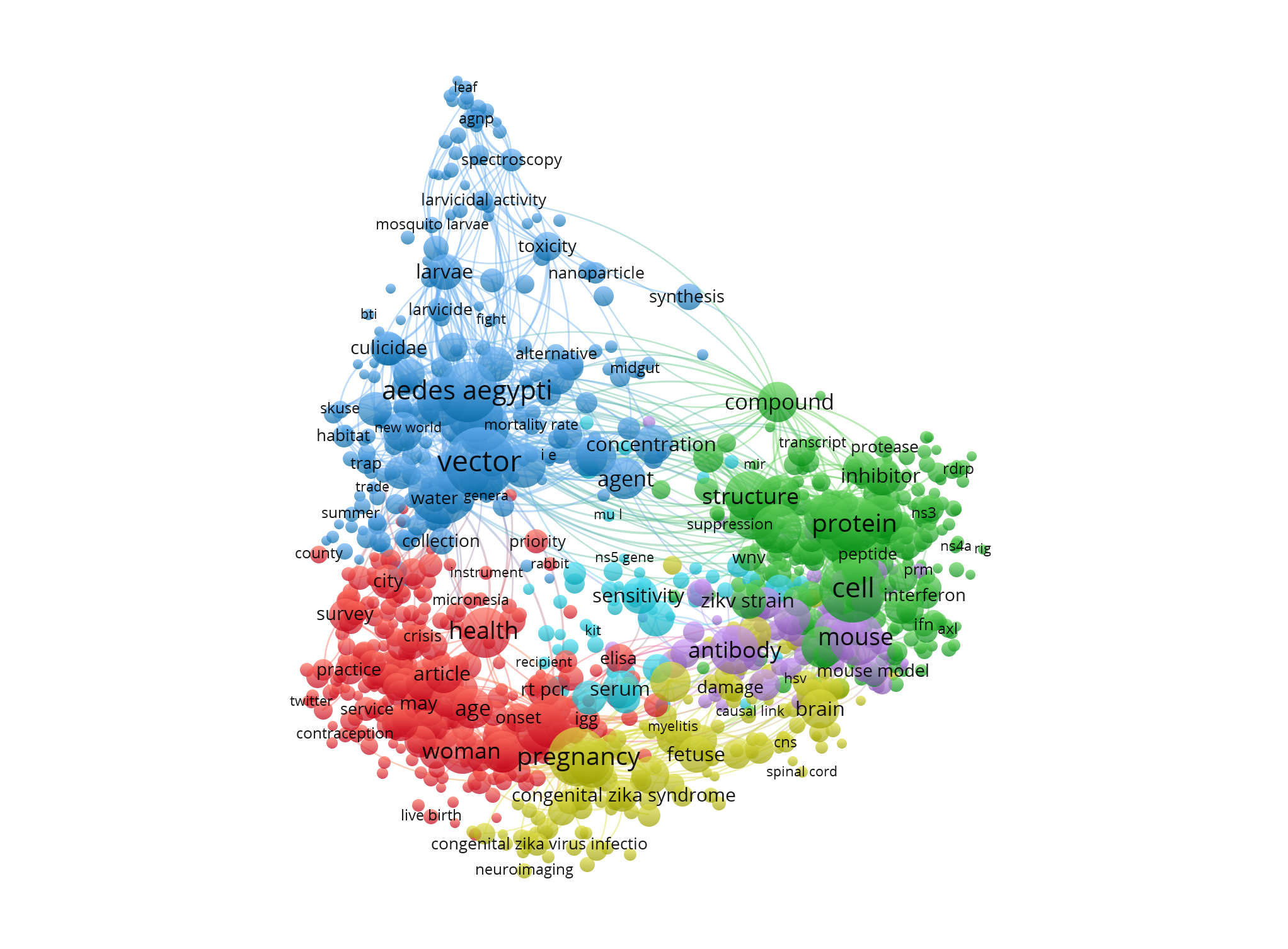

Supplement: S3 Fig — (TIF) [file pone.0229790.s003.tif]

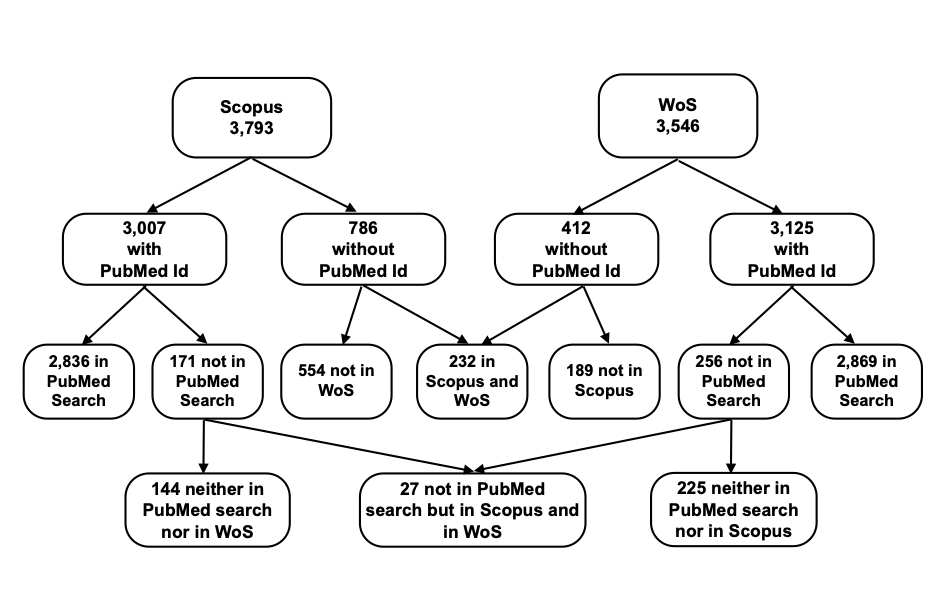

Supplement: S4 Fig — (TIF) [file pone.0229790.s004.tif]
