# Supplementary material for: The global scientific research response to the public health emergency of Zika virus infection
Source: PLoS One. 2020 Mar 12;15(3):e0229790. doi: 10.1371/journal.pone.0229790 (PMC7067462; doi:10.1371/journal.pone.0229790)
Supplement: S1 File — (DOCX) [file pone.0229790.s006.docx]

**The global scientific research response to the public health emergency of Zika virus infection**

**Supplementary Information**

Juliane Fonseca de Oliveira^1,2^, Julia Moreira Pescarini^1^, Moreno de Souza Rodrigues^1,3^, Bethania de Araujo Almeida^1^, Claudio Maierovitch Pessanha Henriques^4^, Fabio Castro Gouveia^5^, Elaine Teixeira Rabello^6,9^, Gustavo Correa Matta^7^, Mauricio L. Barreto^1,8^, Ricardo Barros Sampaio^1,4^

^1^ Centro de Integração de Dados e Conhecimentos para Saúde (Cidacs), Instituto Gonçalo Moniz, Fiocruz, Salvador, Bahia, Brazil, ^2^ Centro de Matemática da University of Porto, Porto, Portugal, ^3^ Laboratório de Análise e Visualização de dados, Fiocruz, Rondônia, Brazil, ^4^ Gerência Regional de Brasília, Fiocruz, Brasília, Brazil, ^5^ Casa Oswaldo Cruz, Fiocruz, Rio de Janeiro, Brazil, ^6^ Instituto de Medicina Social, Universidade do Estado do Rio de Janeiro, Rio de Janeiro, Brazil, ^7^ Escola Nacional de Saúde Pública, Fiocruz, Rio de Janeiro, Brazil, ^8^ Instituto de Saúde Coletiva, Universidade Federal da Bahia, Salvador, Bahia, Brazil, ^9^ Public Administration and Policy Group, Wageningen University & Research, Wageningen, The Netherlands

Corresponding author: Ricardo Barros Sampaio and Juliane Fonseca de Oliveira

**S1. File. Description of the data used in the manuscript.**

All data in this manuscript are presented in a single zip file named data.zip file.

- The articles downloaded from the WoS, Scopus and PubMed databases are named as articles_WoS.zip, articles_scopus.zip and articles_pubmed.zip. Using the VOSviewer software, we produced the plots presented in Supplementary figure 1 to 4.
- The descriptive analysis on the countries collaborations and the textual analysis were performed both directly by filtering information from the Web of Science homepage and with the data contained in each article downloaded from the WoS database. Using the VOSviewer software, one can load the information of the articles downloaded from the WoS database and export the “selected_countries” and “(country)author_relationship” files. From the “selected_countries” file we selected the columns with the “id”, “name of the institutional affiliation country”, and the number of documents “produced by the country.'' The “authorship_relation” file gives the countries related to each document for each country appear as co-authors' affiliation countries . The total number of authors that are co-author in an article is also informed in the VOSviewer. These files were used to construct Table 1 and Fig 2 in the manuscript. We provide the “selected_countries”, “(country)author_relationship” files in the data.zip file.
- The information in Fig 3 in the manuscript was produced by filtering information from the Web of Science homepage. We included the data_Fig3.csv file with the information we collected to the end of December 2018.
- Fig 4 is produced in the VOSviewer after loading the information of the articles downloaded from the WoS database.
- The input data for IRAMuTeQ used to describe the main areas of knowledge are presented in the file ira_zika.txt. The remaining plots in the manuscript are then produced using IRAMuTeQ.
