# Supplementary material for: The global scientific research response to the public health emergency of Zika virus infection
Source: PLoS One. 2020 Mar 12;15(3):e0229790. doi: 10.1371/journal.pone.0229790 (PMC7067462; doi:10.1371/journal.pone.0229790)
Supplement: Attachment — Submitted filename: Rebuttal_Letter_17_12_2019.docx [file pone.0229790.s007.docx]

Dear Editor and Reviewers,

With this letter, we resubmit the paper entitled “**The global scientific research response to the public health emergency of Zika virus infection**” (PONE-D-19-19413) for review to PLOS One after incorporating the comments from the reviewer and editor. The text has been fully revised taking into account the comments and suggestions made by the reviewer and editor. We answered all questions and incorporate all the suggestions, which we believe has made the manuscript more clear and understandable. We hope that you will now find it suitable for publication. Our responses to the reviewers appear on the pages that follow.

We thank you again for the opportunity to submit our revised manuscript for publication consideration. We believe that through addressing the reviewers’ comments, the quality of our paper has improved considerably.

Yours Sincerely,

Ricardo Barros Sampaio

On behalf of all authors.

Comments and replies

**Editor comments**

| **Comment:** “As you will gather from the reviewer comments, the data presented in your manuscript are particularly interesting and offer a wide and global vision on the scientific efforts that were made during the last ZIKV epidemic. I also express this view after careful examination of your study, which I find well suited for the journal.  **1.1:** However, both the reviewer and myself found that a (final) section discussing what can be learned from the general conclusions of your data and the preparation for future epidemic is currently lacking and needed. Specifically, even if poorly provided in data, a discussion on what was perceived by the community as not or poorly effective during the epidemic (e.g. Funding availabilities, timing of international programs, data collecting...) would greatly benefit your manuscript.  **1.2:** Moreover, we note that your search was performed using only one database; to ensure that a thorough search is presented, we would request that you update the search to include at least a second database. | **Reply 1.1:** Agreed. Discussion and Conclusion sections have been modified to incorporate how our findings can contribute to future epidemic responses and preparedness. Also, we improved the Introduction section aiming to clarify our objectives.  **Reply 1.2:** We thank the editor for this comment. In order to account for your suggestion, we performed searches in two additional databases: Scopus and PubMed. Descriptive analysis on the databases WoS, Scopus and PubMed was incorporated in the text and knowledge maps of words extracted from titles and abstracts of articles on WoS, Scopus and PubMed are presented in supplementary figures S1 Fig to S3 Fig. They were obtained using VOSviewer software. We can see that the clusters found from VOSviewer software for the three databases are very similar. This results support our choice to carry out textual analysis only with the WoS database. |
| --- | --- |

**Reviewer #1**

| **Comment 1:** Have the authors made all data underlying the findings in their manuscript fully available?  Reviewer #1: No | **Reply 1:** Thank you for this remark. We have included the data extracted from Wos, Scopus and Pubmed and the additional files created to conduct our analysis as Supplementary Material (S2 File). A description of the data used in the manuscript is presented in the S1 File. |
| --- | --- |
| **Comment 2. Review Comments to the Author.**  Reviewer #1: In this study, authors aimed to verify how the Zika epidemic has molded scientific production worldwide analyzing international collaboration and the knowledge landscape through time, research topics and country involvement. Mapping the response to Zika, a public health emergency, demonstrated a clear pattern of the participation of countries in the scientific advances. The pattern of knowledge production found in this study represented the different perspectives and interests of countries based firstly on their level of exposure to the epidemic and secondly on their financial positions with regard to  Science. The data presented in this study are interesting. They confirm already known results: the leading role of the USA in biomedical research, the strong involvement of countries directly affected, the need to establish collaborations for more basic research. This work is very descriptive.  **2.1:** It does not determine what has worked well and what has not worked in terms of international research and collaboration. This greatly limits the scope of this study.  **2.2:** Indeed, we do not see what lessons could be learned for a future epidemic of this type. | **Reply 2.1:** We believe that the success of the scientific collaboration is hard to measure but the utility of each manuscript, on the other hand, can be partially estimated by its citations. Nevertheless, in this manuscript, due to the limited time frame in which Zika research is available, we opted for not evaluating citation metrics. Please, see also the Reply 1.1 for the editor comment.  **Reply 2.2:** Please, see Reply 1.1 for the editor comment. |
